# Microbial Competition and Nutrient Limitation Remodel the Volatilome of *Kluyveromyces marxianus*

**DOI:** 10.3390/jof12070470

**Published:** 2026-06-25

**Authors:** Erick D. Acosta-García, Jesús B. Páez-Lerma, Martha R. Moreno-Jiménez, Edith Cortés-Barberena, Juan A. Rojas-Contreras, Nicolas O. Soto-Cruz

**Affiliations:** 1 Departamento de Ingenierías Química y Bioquímica, Tecnológico Nacional de México, Instituto Tecnológico de Durango, Blvd. Felipe Pescador 1830 ote., Durango 34080, Mexico; 20040540@itdurango.edu.mx (E.D.A.-G.); jpaez@itdurango.edu.mx (J.B.P.-L.); mrmoreno@itdurango.edu.mx (M.R.M.-J.); jrojas@itdurango.edu.mx (J.A.R.-C.); 2 Departamento de Ciencias de la Salud, División de Ciencias Biológicas y de la Salud, Universidad Autónoma Metropolitana, Ciudad de México 09340, Mexico; cobe@xanum.uam.mx

**Keywords:** non-*Saccharomyces* yeast, yeast-yeast competition, volatile fatty acids

## Abstract

The use of *Kluyveromyces marxianus* in mixed cultures for fermentation processes has become increasingly relevant. This yeast is characterized by rapid growth, thermotolerance, broad sugar utilization, and the ability to produce aroma-active compounds. In this study, we evaluated changes in the growth and volatilome of a *K. marxianus* strain isolated from agave fermentation under microbial competition induced by co-cultivation interactions and nutritional limitation induced by a nutrient-deficient medium. The results indicate that these stress factors are significant drivers of metabolic changes, leading to substantial increases in the concentrations of key aromatic compounds. Stress-free conditions favor cell growth and the production of stable, reproducible volatile profiles, which is advantageous for batch-to-batch consistency (as in wine or mezcal production). While microbial competition and nutritional limitation induce reduced cell growth and loss of viability, they also lead to increased aromatic diversity, particularly the synthesis of β-phenethyl acetate, ethyl octanoate, and ethyl hexanoate. These findings demonstrate a relationship between environmental stress and the development of volatile profile complexity, offering new insights into harnessing stress-induced changes in the volatilome to optimize the sensory profile of traditional fermentations.

## 1. Introduction

Microbial diversity, inherent to natural fermentation processes, plays a crucial role in the sensory characteristics of the final product [1]. In recent years, there has been growing interest in mixed fermentations, including non-*Saccharomyces* yeasts, due to their ability to enhance aroma profiles and increase the complexity of fermented beverages [2]. Among these, *Kluyveromyces marxianus* stands out for its ability to utilize a wide range of sugars, such as lactose and inulin [3]. This yeast is thermotolerant, secretes lytic enzymes, and exhibits a high growth rate, making it a promising candidate for biotechnological applications [4,5]. Depending on its origin, *K. marxianus* exhibits high phenotypic variability. For instance, strains isolated from the agave fermentation process show increased tolerance to ethanol and saponins, in some cases achieving higher ethanol yields than *S. cerevisiae* and producing a greater diversity of volatile compounds [6].

The combined use of *Saccharomyces* and non-*Saccharomyces* yeasts contributes to aromatic diversity in fermentation processes and improves organoleptic characteristics of the final product [7]. However, interactions among these yeasts, including mutualism, commensalism, amensalism, or competition, are not controlled, leading to variability in fermentation outcomes. These interactions shape the structure and dynamics of microbial communities [8].

During mixed fermentations, yeasts may experience abiotic, biotic, or combined stress conditions. Physicochemical factors, including temperature, pH, radiation, nutrient availability, and toxic compounds, cause abiotic stress. In contrast, biotic stress is mediated by interactions among microbial populations [9]. The effects of abiotic stress have been widely studied, often by exposing yeasts to specific stressors to modulate metabolic outputs, such as modifying microbial lipid production [10] or improving biofuel yields [11]. For example, ethanol-induced stress in species such as *Pichia anomala* can affect fundamental processes, including ABC transporters, energy metabolism, and carbohydrate metabolism [12]. Other stressors, such as O_3_, H_2_O_2_, and CO_2_, can redirect metabolic flux from glycolysis toward the pentose phosphate pathway and inhibit the aspartate pathway for lysine synthesis, mechanisms that contribute to NADPH generation and the maintenance of cellular antioxidant capacity [13]. Recently, Acosta-Cuevas et al. [14] evaluated the effects of ethanol, isoamyl acetate, acetic acid, and hydrogen peroxide and reported an increase in isoamyl acetate production by *K. marxianus* under hydrogen peroxide-induced oxidative stress. Similarly, exposure to toxic compounds such as lipopolysaccharides can inhibit oxidative phosphorylation, triggering oxidative stress, decreased growth, and cell wall remodeling [15]. Alterations in the volatilome result from all these metabolic changes, representing an attempt by the yeast to maintain homeostasis or facilitate detoxification [16]. Causing the production of ethyl esters such as ethyl acetate and ethyl propionate [13], increasing alcohols and aldehydes [15], and accumulating isovaleric acid, acetic acid, and higher alcohols [17].

In contrast, biotic stress during fermentation can significantly alter volatile compound production. Co-cultivation of *Saccharomyces cerevisiae* with non-*Saccharomyces* yeasts, such as *Metschnikowia pulcherrima*, *Torulaspora delbrueckii*, or *Hanseniaspora*, has been shown to produce volatile profiles that differ markedly from those observed in pure cultures [18,19]. Mixed fermentations involving *Hanseniaspora vinae* and *Torulaspora delbrueckii* have been associated with increased ester production and reduced acetic acid levels [20]. Li et al. [21] reported that, in monocultures, the number of volatile compounds increased progressively throughout fermentation. However, in mixed cultures, volatile compound levels initially increased and subsequently decreased. This behavior was attributed to the early dominance of *P. kluyveri*, followed by its inhibition and replacement by *S. cerevisiae*, which utilized these compounds as precursors for volatile compounds. More recently, Acosta-García et al. [22] reported a marked loss of viability of *Torulaspora delbrueckii* in co-culture with *Saccharomyces cerevisiae* after 12 h of incubation in M2 medium. In contrast, no such effect was observed in YPD medium. These findings suggest that the nutritional richness of the culture medium modulates biotic stress.

Given the relevant fermentative characteristics of *K. marxianus*, this study aimed to evaluate the effects of yeast-yeast competition and nutrient limitations on fermentation kinetics and the volatilome of *K. marxianus* in co-culture with *S. cerevisiae* and *T. delbrueckii*. Understanding how stress conditions influence the volatilome of *K. marxianus* will help optimize its use in fermentation processes, whether to maintain consistent, reproducible aroma profiles or to enhance aromatic diversity and product complexity.

## 2. Materials and Methods

### 2.1. Strains and Inoculum Preparation

The strains *Saccharomyces cerevisiae* ITD-00185, *Kluyveromyces marxianus* ITD-01005, and *Torulaspora delbrueckii*, previously isolated from mezcal production, were used in this study [23,24,25]. The inoculum was prepared from a single colony of each yeast strain suspended in 50 mL of liquid YPD medium (2% glucose, 2% casein peptone, and 1% yeast extract) and incubated for 12 h at 28 °C with agitation at 150 rpm in an orbital shaker (Shel Lab model SSI2, Shel Lab, Cornelius, OR, USA).

### 2.2. Monocultures and Co-Cultures

Fermentations were carried out in two culture media with different nutritional qualities: YPD and M2. The YPD medium contained (per liter): fructose, 90 g; glucose, 10 g; yeast extract, 10 g; casein peptone, 20 g. The M2 medium [26] contained (per liter): fructose, 90 g; glucose, 10 g; yeast extract, 1 g; (NH_4_)_2_SO_4_, 2 g; MgSO_4_•7H_2_O, 0.4 g; KH_2_PO_4_, 5 g. The pH of the M2 media was adjusted to 5. All fermentations were performed in triplicate using 150 mL of medium, incubated at 28 °C and 150 rpm in an orbital shaker (Shel Lab model SSI2). Monocultures were initiated at a cell density of 1 × 10^6^ cells/mL. Binary co-cultures were initiated at 5 × 10^5^ cells/mL for each strain, and ternary co-cultures at 3.3 × 10^5^ cells/mL for each strain. This initial density ensures that nutrient availability per cell remains uniform at the start of fermentation. Fermentations were monitored every 3 h for 24 h. Samples were collected in triplicate to determine the growth of each yeast species, as well as glucose and fructose consumption and ethanol production. Each sample was centrifuged at 6720× *g* for 3 min, and the supernatant was filtered through a 0.45 µm Nylon membrane. Filtrates were stored at −20 °C until analysis. The volatilome was determined from the residual medium after 24 h of incubation.

### 2.3. Analytical Techniques

#### 2.3.1. Yeast Growth

Population growth was determined by viable cell counting. For monoculture samples, MGYP agar plates (glucose, 10 g/L; casein peptone, 5 g/L; malt extract, 3 g/L; yeast extract, 3 g/L; agar, 20 g/L) were used. For co-culture samples, Wallerstein Laboratory Nutrient Agar (MilliporeSigma, Billerica, MA, USA) was employed, which allows differentiation of yeast species based on morphological characteristics.

#### 2.3.2. Sugar Consumption and Ethanol Production

Glucose and fructose consumption, as well as ethanol production in the fermentation samples, were determined by high-performance liquid chromatography using an Agilent 1200 series HPLC system (Agilent Technologies Inc., Santa Clara, CA, USA). The chromatograph was equipped with an Aminex HPX-87H column (300 mm × 7.8 mm, Bio-rad Laboratories, Inc., Hercules, CA, USA), and 5 mM H_2_SO_4_ was used as the mobile phase at a flow rate of 0.6 mL/min. The oven temperature was maintained at 60 °C, and detection was performed using a refractive index detector. The detector temperature was set to 45 °C. Calibration curves were constructed using external standards for quantification. HPLC-grade glucose and fructose were purchased from Sigma-Aldrich (St. Louis, MO, USA) with purities ≥ 99%. Ethanol from CTR Scientific (Guadalajara, Mexico) with a purity of ≥99.5%. One microliter of each appropriately diluted sample was injected, and all samples were analyzed in triplicate.

#### 2.3.3. Identification and Semi-Quantification of Volatile Compounds

The volatile compounds present at the end of fermentations (24 h incubation) were identified using HS-SPME/GC-MS, following the procedure described by Acosta-García et al. [22]. Briefly, after centrifugation and filtration, 5 mL of filtrate was transferred to a microextraction flask. Subsequently, 1.5 g of NaCl and 50 µL of a 1-pentanol solution (300 mg/L) were added as an internal standard. The mixture was incubated for 5 min at 35 °C in a water bath. A 50/30 µm DVB/CAR/PDMS fiber (Supelco, St. Louis, MO, USA) was exposed to the headspace for 60 min at 35 °C. After extraction, the fiber was placed in the injection port of an Agilent 7890A gas chromatograph and exposed to 250 °C for 10 min. Compounds were separated using an FFAP column (30 m × 250 mm × 0.25 µm). Ultra-high-purity helium was used as the carrier gas at a flow rate of 1 mL/min. The oven temperature was initially set to 40 °C for 3 min, then ramped from 3 °C to 52 °C for 1 min, and finally ramped from 10 °C to 200 °C for 15 min. The mass spectrometer (Agilent 5975C) was operated at 230 °C, with an ionization voltage of 70 eV, in Scan mode (1.6 scans per second).

The identification of volatile compounds was performed as described by Acosta-García et al. [27]. The AMDIS software (v. 2.73, build 149.31) was used to deconvolve the mass spectra, and the results were compared with the NIST 2011 library using a match value ≥ 80%. In parallel, the retention index [28] was calculated based on a C7–C30 alkane ladder and compared with values reported in the NIST database (https://webbook.nist.gov/chemistry/name-ser/, accessed on 30 march 2026).

The semi-quantification of volatile compounds was performed based on the mass of the internal standard [29] using the equation:

$$C_{x}=C_{i}\times \frac{A_{x}}{A_{i}}$$

where $C_{x}$ corresponds to the concentration of the unknown analyte (mg/L), $C_{i}$ is the concentration of the internal standard (mg/L), $A_{x}$ is the peak area of the unknown analyte, and $A_{i}$ is the peak area of the internal standard. The concentration obtained using this equation represents a semi-quantitative estimate based on the response relative to the internal standard. It should not be interpreted as an absolute concentration. Then, the abundance of each compound was expressed as the equivalent mass relative to 1 µg/L of 1-pentanol, used as the internal standard. Despite the semi-quantitative nature of this approach, it provides a robust basis for comparing metabolite levels and identifying statistically significant differences among experimental conditions.

### 2.4. Relative Olfactory Activity Value (rOAV) Calculation

To evaluate the effects of microbial competition and nutritional limitation on aroma production, odor thresholds, and aroma descriptors for each volatile compound, data were obtained from online databases (https://thegoodscentscompany.com; https://www.femaflavor.org; https://www.flavornet.org) and previous studies [30,31,32,33]. Two levels of odor activity values were calculated: rOAV to determine individual potency based on detection thresholds, and ROAV to establish a hierarchy of aromatic importance. While rOAV is a direct ratio, ROAV represents a normalization in which the most influential compound in the mixture is scaled to 100. The rOAVs and ROAVs values were calculated according to Jiang et al. [34], using the following equations:

$$rOAV_{i}=C_{i}/{OT}_{i}$$

$$ROAV_{i}={rOAV}_{i}/{rOAV}_{max}\times 100$$

where $rOAV_{i}$ is the relative olfactory activity of compound *i* (dimensionless),

$C_{i}$ is the relative concentration of compound *i* (mg/L),

${OT}_{i}$ is the concentration of compound *i* corresponding to its detection threshold (mg/L),

$ROAV_{i}$ is the relative olfactory activity of compound *i* normalized with respect to the compound present in the highest concentration in the sample (dimensionless),

${rOAV}_{max}$ is the maximum rOAV value recorded in the sample, representing the compound with the greatest aromatic impact (dimensionless) [34].

### 2.5. Statistical Analysis

Multivariate statistical analyses were performed to determine key volatile compounds in the fermentation. Principal component analysis (PCA), orthogonal partial least squares discriminant analysis (OPLS-DA), base-2 logarithm of the fold change (Log_2_(FC)), and variable importance in projection (VIP) were performed using MetaboAnalyst 6.0 (https://www.metaboanalyst.ca/). Data were normalized by sum and scaled by centering the mean and dividing by each variable’s standard deviation. To discriminate key compounds, monocultures were matched with their respective co-cultures, and compounds with VIP ≥ 1, Log_2_(FC) ≥ 1, and *p*-value ≤ 0.05 were identified [30].

## 3. Results and Discussion

This study focused on how the combination of two types of stress (nutritional limitation and microbial competition) affects the growth and volatile compound production of *Kluyveromyces marxianus*. Nutritional limitation was studied using two culture media: (1) M2 medium, designed to simulate the characteristics of diluted agave juice and considered nutritionally limited, and (2) a modified YPD medium, regarded as nutritionally rich. Compared with the nutrient-rich YPD medium, M2 represents a more restrictive nutritional environment. The 10-fold reduction in yeast extract and the complete omission of casein peptone severely limit the availability of complex organic nitrogen, free amino acids, vitamins, and trace elements, thereby establishing a controlled model for nutritional limitation. Microbial competition was induced via co-cultivation in both binary and ternary combinations with *Saccharomyces cerevisiae* and *Torulaspora delbrueckii*. Monocultures of each yeast strain were performed as controls.

### 3.1. Effect of Microbial Competition and Nutritional Limitation on the Growth of K. marxianus

**Figure 1:**
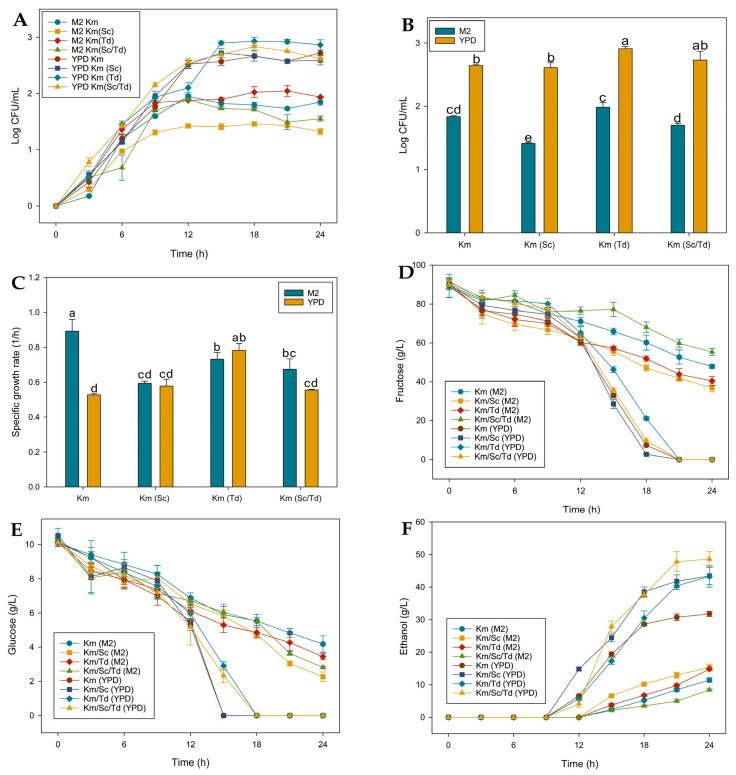
(**A**) Fermentation kinetics for monocultures and co-cultures. (**B**) Maximum growth of yeasts in monoculture and co-culture. (**C**) Specific growth rate of yeast in monoculture and co-culture. (**D**) Fructose consumption. (**E**) Glucose consumption. (**F**) Ethanol production. Data are shown as the average of three replicates, and error bars were obtained from standard deviations. Different letters indicate significant differences (Tukey test, *p* < 0.05).

Figure 1A shows the growth of *K. marxianus* in monoculture and co-culture in YPD and M2 media. In YPD medium, all cultures produced more biomass (Figure 1A) than in M2 medium. The M2 medium supported growth comparable to YPD only during the initial 6 h of incubation. After 9 h, *K. marxianus* monocultures exhibited higher biomass than co-cultures, where growth was clearly reduced. In Figure 1B, it can be observed that the presence of *S. cerevisiae* reduces the maximum growth (*X_max_*); this is notable in the M2 medium, where the cultures in which *S. cerevisiae* participated obtained a lower cell density (Km/Sc = 1.414 ± 0.012 Log CFU/mL and Km/Sc/Td = 1.701 ± 0.031 Log CFU/mL) than the *K. marxianus* monoculture (1.837 ± 0.015 Log CFU/mL). This effect is likely associated with yeast-yeast competition under nutrient-limited conditions.

**Figure 2:**
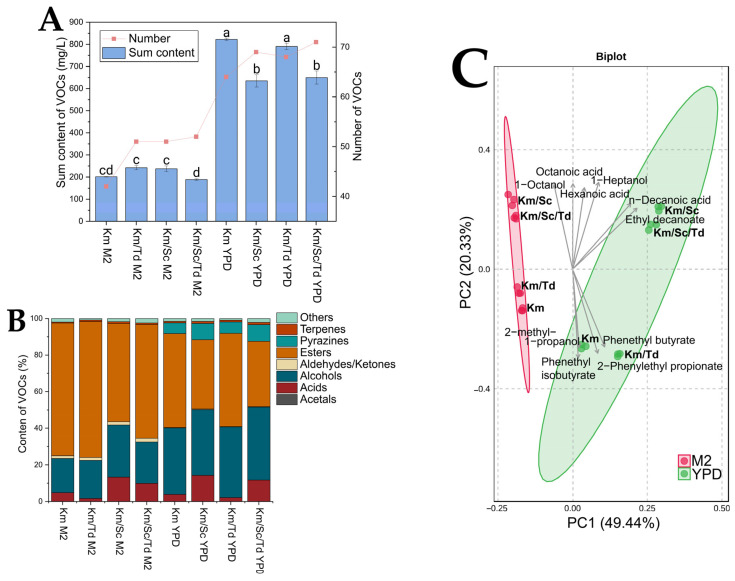
Total concentration and number of compounds produced (**A**), distribution by chemical families (**B**), and main component analysis (**C**) of volatile compounds at 24 h of fermentation. Different letters indicate significant differences (Tukey test, *p* < 0.05).

In contrast, in YPD medium, the presence of *S. cerevisiae* did not significantly reduce the viability of *K. marxianus* compared to monoculture (Figure 1B). In the nutrient-rich YPD medium, the specific growth rate of *K. marxianus* increased in mixed cultures compared to monocultures (Figure 2C), suggesting a possible metabolic synergy or sequential nutrient utilization when resources are not limited. Conversely, the drastic reduction in biomass and specific growth rate of *K. marxianus* in the M2 medium, particularly in the presence of *S. cerevisiae*, suggests that organic nutrient restriction intensifies competitive exclusion.

The inhibitory effect of *S. cerevisiae* on *K. marxianus* has been previously observed in citrons [35], synthetic media [36], and agave juice [37]. However, other studies have shown that *K. marxianus* can coexist or even benefit from co-cultivation with *S. cerevisiae*. For instance, mixed cultures containing immobilized cells of *K. marxianus* and *S. cerevisiae* yielded higher ethanol than monocultures [38]. Additionally, *K. marxianus* has been reported to secrete lytic enzymes that reduce the viability of *S. cerevisiae* [39].

In contrast to the interaction with *S. cerevisiae*, *K. marxianus* exhibited improved growth when co-cultured with *T. delbrueckii* in both media (Figure 1B). Although competition among non-*Saccharomyces* species is generally considered less intense [8], mixed cultures have been associated with improved sensory quality of fermented beverages [40].

In the ternary consortium within medium M2, *K. marxianus* maintained greater viability than in the binary culture with *S. cerevisiae*. Although we cannot definitively confirm the mechanisms without direct analysis of metabolic flux, this partial rescue effect could be attributed to niche overlap or to *K. marxianus* utilizing metabolites released upon the death of *T. delbrueckii*. Metabolic exchange among microbial species is a common phenomenon [41], involving the exchange of amino acids, nucleotides, and other metabolites, thereby supporting the survival of certain species within a consortium [42].

Phylogenetic distance has been proposed to influence interspecies interactions [43]. Closely related species often exhibit similar nutrient consumption patterns, which may intensify competition [44]. *Saccharomyces cerevisiae* and *T. delbrueckii* are phylogenetically more closely related to each other than to *K. marxianus* [43]. This relationship may explain the stronger inhibitory interactions between *S. cerevisiae* and *T. delbrueckii*, as well as the lower competition between *S. cerevisiae* and *K. marxianus*. However, Pourcelot et al. [45] emphasize that additional mechanisms also contribute to these interactions, concluding that the species determines the type of interaction, while the strain modulates its intensity.

Figure 1D–F illustrate glucose and fructose consumption and ethanol production. Notably, none of the fermentations in M2 medium achieved complete sugar consumption. Under these conditions, the ternary co-culture exhibited the highest residual fructose concentration (55.37 ± 1.80 g/L; Figure 1D), whereas the *K. marxianus* monoculture showed the highest residual glucose concentration (4.17 ± 0.48 g/L; Figure 1E). These differences were reflected in ethanol production (Figure 1F). The co-culture of *K. marxianus* with *S. cerevisiae* yielded the highest ethanol production (15.32 ± 1.00 g/L). In contrast, the ternary co-culture produced the lowest ethanol content (8.45 ± 0.30 g/L) in M2 medium. In contrast, all fermentations in YPD medium resulted in complete sugar consumption. Glucose was depleted at approximately 15 h, whereas fructose remained detectable until around 18 h. Consequently, ethanol production was higher under these conditions. Unlike the results observed in M2 medium, the ternary co-culture in YPD produced the highest ethanol concentration (48.60 ± 2.32 g/L). Similarly, Nolasco-Cancino et al. [46] reported that *K. marxianus* monocultures consumed up to 90% of sugars in agave juice after 72 h, and that the presence of *S. cerevisiae* improved ethanol production efficiency.

Overall, the results shown in Figure 1 indicate that *K. marxianus* can tolerate individual stress conditions, whether competition or nutritional. However, the combination of both stress types resulted in a reduction in cell viability (~20%) after 9 h of cultivation. This effect may be attributed to the limited nutrient content of the M2 medium, rather than solely to nitrogen availability, as micronutrients such as zinc and magnesium are essential for efficient fermentation [47]. However, the use of synthetic media presents significant limitations, as they do not fully replicate the complexity of industrial fermentation environments. Natural substrates, such as agave juice, grape must, or other fermentation matrices, contain diverse sources of carbon and nitrogen, micronutrients, inhibitory compounds, and indigenous microbial communities that can influence both population dynamics and the production of volatile compounds.

### 3.2. Volatilome Produced by K. marxianus

A total of ninety volatile organic compounds (VOCs) were identified (Table S1), including 1 acetal, 7 acids, 15 alcohols, 7 ketones and aldehydes, 28 esters, 9 pyrazines, 13 terpenes, and 10 miscellaneous VOCs (furans, phenols, methyl sulfide, lactone, hydroxy ester, and pyrrole). Chromatographic peaks corresponding to nonanoic acid, 1-propanol, ethyl butyrate, carvacrol, and thymol overlapped in some samples (Table S2). They were therefore excluded from the statistical analysis. Volatile compounds were analyzed at 24 h of culture. While this single-time-point approach does not capture the kinetic variations in metabolite production and consumption throughout the entire fermentation, it was selected because the volatilome at the end of alcoholic fermentation is the most relevant.

Fermentations carried out in medium M2 showed both a lower number and a lower total concentration of VOCs (Figure 2A) than those performed in YPD medium. The ternary co-culture in M2 medium showed the lowest total VOC content (188.76 ± 3.55 mg/L). In contrast, the *K. marxianus* monoculture and the *K. marxianus*/*T. delbrueckii* co-culture in YPD medium displayed the highest VOCs concentrations (822.04 ± 5.77 mg/L and 790.41 ± 14.23 mg/L, respectively).

Figure 2B shows the distribution of VOCs by chemical family. Fermentations in YPD medium showed a higher proportion of alcohols (35–40%), whereas in M2 medium this fraction was lower (18–28.5%). Alcohols production via the Ehrlich pathway contributes to NADH regeneration [48], thereby helping maintain intracellular redox balance. In this pathway, α-Ketoacid decarboxylases catalyze the enzymatic degradation of amino acid-derived α-keto acids into aldehydes, which are subsequently reduced to fusel alcohols or oxidized to fusel acids [44]. Thus, the higher alcohol content observed in YPD fermentations is consistent with previous reports suggesting that increased amino acid availability may enhance higher alcohol production via the Ehrlich pathway.

Nutritional limitations in M2 medium may redirect metabolic flux toward ester production as a strategy to mitigate alcohol-induced stress. Accordingly, fermentations in M2 medium yielded a higher proportion of esters (62–72.5%), whereas fermentations in YPD yielded lower ester levels (38–51.5%). This difference may be linked to stress conditions in M2 medium, as increased ester synthesis has been associated with the regeneration of free CoA and the detoxification of the fermentation medium [49].

On the other hand, the presence of *S. cerevisiae* resulted in a higher proportion of organic acids (9–14.5%) than in fermentations involving only non-*Saccharomyces* yeasts, where levels were below 4%. This observation suggests that *S. cerevisiae* may employ acid production as a competitive strategy. Increased organic acid concentrations have been reported to increase membrane permeability and impair cellular metabolism in competing yeasts [50,51].

The presence of aldehydes and ketones is particularly relevant, as these compounds have been reported to exert both positive and negative sensory effects. Some aldehydes contribute pleasant, fruity, and floral notes. In contrast, others impart green descriptors that may be considered undesirable depending on their concentration and the product matrix [51,52]. Fermentations in M2 medium showed concentrations ranging from 1.4–2.1%, while in YPD medium, these compounds remained below 0.33% in all fermentations. Pyrazines were detected exclusively in YPD fermentations, potentially originating from yeast extract components present in the culture medium [53].

In contrast, terpene content remained relatively consistent across all samples. Overall, these results suggest that M2 medium provides a more favorable compositional profile for generating a desirable aroma characteristic, owing to its higher ester content and lower alcohol content, despite exhibiting a lower total concentration and diversity of VOCs (likely associated with reduced sugar consumption). However, the presence of aldehydes and ketones in M2 fermentations may negatively affect overall aroma perception. Nevertheless, the impact of these compounds on the final product requires further investigation.

The 2D PCA plot (Figure 2C) shows the proximity of the replicates, indicating analytical robustness, with the first two principal components explaining 69.7% of the total variance. A clear separation between the two media used (M2 and YPD) was observed. Based on variable loadings, the VOCs contributing most strongly to this separation included: octanoic acid, hexanoic acid, n-decanoic acid, 1-octanol, 1-heptanol, ethyl decanoate, 2-methyl-1-propanol, phenethyl butyrate, phenethyl isobutyrate, and 2-phenylethyl propionate.

2-Phenylethyl propionate and phenethyl butyrate were detected exclusively in the monoculture of *K. marxianus* and *T. delbrueckii*, as well as in their co-cultures. In contrast, phenethyl isobutyrate was identified only in the monocultures *K. marxianus* and its co-cultures Phenethyl butyrate reached higher concentrations in YPD fermentations, particularly in *K. marxianus* monoculture (24.058 ± 0.563 mg/L) and in co-culture with *T. delbrueckii* (21.831 ± 0.424 mg/L). In contrast, co-culture with *S. cerevisiae* significantly reduced the phenethyl butyrate concentration (3.507 ± 0.562 mg/L). The presence of phenethyl butyrate has been reported previously in cheeses fermented with *Kluveromyces latis*, both in monoculture and in co-culture with *T. delbrueckii* [54,55].

### 3.3. Aromatic Contribution of K. marxianus Under Nutritional Limitation and Microbial Competition

Odor activity value (OAV) analysis is a widely used metric for assessing aroma potential, accounting for the equilibrium between the food matrix and the gas phase. Compounds with higher OAVs contribute more significantly to overall aroma complexity [56]. However, this approach should be verified by sensory evaluation. Volatile compounds with a relative ROAV ≥ 1 are considered key aroma contributors. Conversely, those with ROAVs between 0.1 and 1 are considered secondary contributors that indirectly contribute to aroma complexity. A total of 54 compounds with a ROAV ≥ 0.1 were identified across all fermentations (Table S3), of which 33 showed ROAV ≥ 0.1, and 13 exhibited ROAV ≥ 1.

A wide range of aroma descriptors was observed. Compounds were grouped into seven main categories based on aromatic similarity: (1) fruity (aromas associated with fresh or exotic fruits); (2) sweet (notes derived from toasted, baked or dairy sugars); (3) Floral (notes reminiscent of flowers); (4) green (vegetal, fresh aromas); (5) fermented (compounds linked to fermentation processes or high volatility); (6) aromatic/woody (resinous aromas or with spicy touches) and (7) lipid-like (aromas related to meats, fats or waxes).

**Figure 3:**
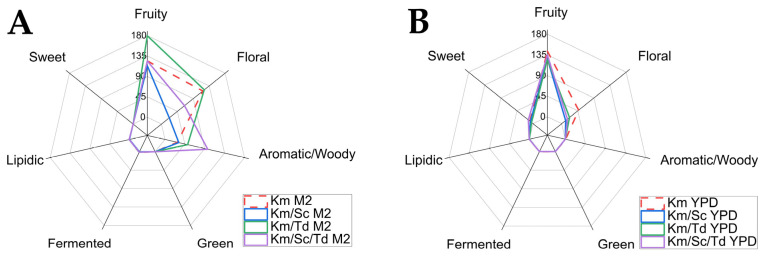

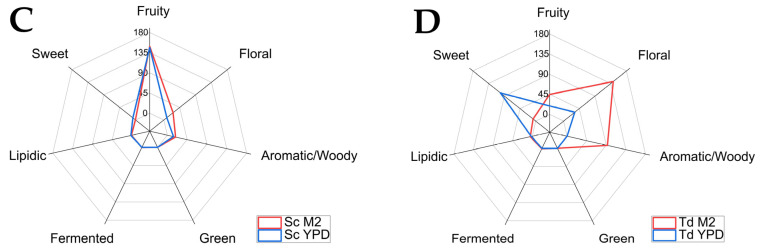
Aromatic profile based on ROAVs for monocultures of *K. marxianus* and its co-cultures in M2 (**A**) and YPD (**B**) media against the aromatic profiles of the monocultures of *S. cerevisiae* (**C**) and *T. delbrueckii* (**D**) from data published by Acosta-García et al. [22].

Figure 3A shows that the aroma profile of *K. marxianus* monoculture in M2 medium was characterized by fruity and floral notes, mainly driven by β-phenethyl acetate (floral; ROAV = 100) and ethyl octanoate (sweet, floral, fruity, banana, pear, brandy; ROAV = 73.01), which were the main contributors to aroma complexity. In contrast, the aroma profile of the *K. marxianus* monoculture in YPD medium (Figure 3B) was dominated by fruity notes, mainly influenced by ethyl octanoate (ROAV = 100) and ethyl hexanoate (fruity, green apple, banana, brandy, wine-like; ROAV = 27.87). Ethyl esters such as ethyl octanoate and ethyl hexanoate have very low sensory thresholds, which explains their consistently high OAVs in fermented beverages such as wine and beer [57]. Additionally, β-phenethyl acetate has been reported as a key aroma contributor in rice wine [58].

Co-cultures in M2 medium (Figure 3A) developed diverse aromatic profiles, predominantly influenced by fruity, floral, and aromatic/woody notes. The *K. marxianus*/*T. delbrueckii* co-culture exhibited a higher intensity of fruity and floral notes, with ethyl hexanoate (ROAV = 80.06) as the main contributor to fruity notes, and β-phenethyl acetate (ROAV = 94.27) contributing to green/floral aroma. In contrast, the *K. marxianus*/*S. cerevisiae* co-culture exhibited the lowest overall aroma intensity, likely due to reduced *K. marxianus* viability in M2 medium. The ternary co-culture exhibited an aroma profile strongly influenced by aromatic/woody notes, particularly due to methyl benzoate (ROAV = 72.03), a phenolic compound with sensory attributes reminiscent of wintergreen essential oil [30]. Although methyl benzoate was also detected in the *K. marxianus*/*S. cerevisiae* co-culture (ROAV = 20.35), its contribution was higher in the ternary co-culture. This compound has been identified as a key contributor to the aroma of jasmine tea [59].

The aroma profiles of the *S. cerevisiae* (Figure 3C) and *T. delbrueckii* (Figure 3D) monocultures were previously reported by our research group [22] and were obtained under the same experimental conditions. The aroma profile of *S. cerevisiae* was consistent across both culture media. It closely resembled the profiles observed in co-cultures grown in YPD. In contrast, *T. delbrueckii* exhibited distinct volatilome profiles depending on the culture medium. This yeast appeared to directly influence the volatilome of the *K. marxianus/T. delbrueckii* co-culture, particularly by producing decanal. This compound was the main contributor to aromatic/woody aroma in both systems (ROAV = 87.06 and 38.32, respectively) and has been described as a characteristic component of the *T. delbrueckii* volatilome [60].

Overall, these results suggest that both microbial competition and nutritional limitation significantly influence the aromatic expression of yeasts. Under favorable growth conditions, such as in nutrient-rich YPD medium, aroma profiles remain relatively stable and similar across fermentations. This stability may enhance batch-to-batch reproducibility (as in wine or mezcal production). However, it may also limit the aromatic diversity contributed by non-*Saccharomyces* species.

### 3.4. Key Volatile Compounds Produced by K. marxianus

Orthogonal partial least squares discriminant analysis (OPLS-DA) was applied to identify key volatile compounds and to evaluate their responses to microbial competition under both culture conditions. Comparisons were performed between *K. marxianus* monoculture and its co-cultures with *T. delbrueckii*, *S. cerevisiae*, and the ternary system *K. marxianus*/*S. cerevisiae*/*T. delbrueckii*. All OPLS-DA models were constructed using a predictive component and an orthogonal component. All models achieved R^2^Y and Q^2^ values greater than 0.8 and 0.7, respectively, indicating good discrimination and predictive ability (Figure S1). Model validation was performed using a permutation test (n = 1000). The frequency distribution showed that all randomly permuted models underperformed the original model, with empirical significance values of *p* ≤ 0.006 for R^2^Y and *p* ≤ 0.006 for Q^2^. These results confirm the model’s statistical validity and rule out overfitting (Figure S2).

**Figure 4:**
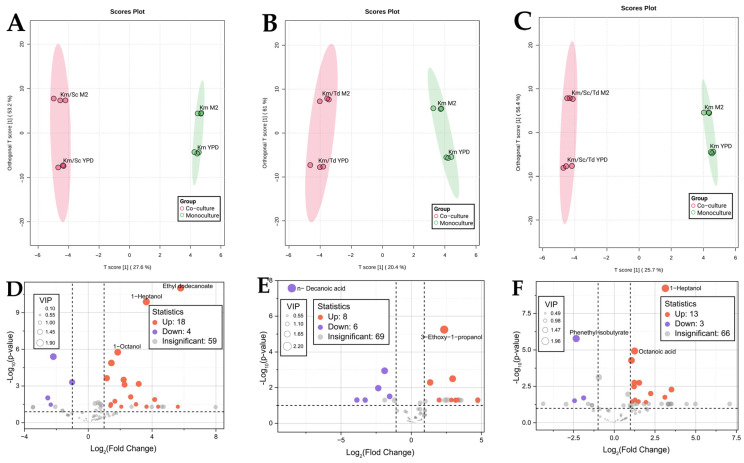
OPLS-DA analysis and volcanic graphs of *Kluyveromyces marxianus* (Km) monoculture versus its co-cultures with *Saccharomyces cerevisiae* (Sc) and *Torulaspora delbrueckii* (Td). OPLS-DA analysis of Km vs. Km/Sc (**A**), Km vs. Km/Td (**B**), and Km vs. Km/Sc/Td (**C**). Volcanic graphs of differential metabolites of Km vs. Km/Sc (**D**), Km vs. Km/Td (**E**), and Km vs. Km/Sc/Td (**F**). Each point on the volcanic graph corresponds to a metabolite, and its size indicates the VIP value.

Score plots (Figure 4A–C) show a clear separation into two distinct groups: monocultures and co-cultures, regardless of the culture medium. It indicates that interspecies interactions have a stronger impact on the volatile profile of *K. marxianus* than on the composition of the culture medium itself.

Key volatile compounds affected by microbial competition, regardless of the culture medium, were examined using the criteria: VIP ≥ 1, |Log_2_(FC)| ≥ 1, and *p* < 0.05. Differences in metabolite abundance between monoculture and co-culture are shown in Figure 4D–F. Blue points represent metabolites downregulated in co-culture, red points indicate metabolites upregulated, and gray points indicate identified compounds with no significant change. Fermentations involving *S. cerevisiae* (Figure 4D,F, Tables S4 and S6) exhibited the highest number of upregulated compounds (18 compounds in Km/Sc and 13 in Km/Sc/Td). In contrast, fermentations involving *T. delbrueckii* (Figure 4E, Table S5) showed only 8 upregulated compounds. These results suggest that competition from *S. cerevisiae* may stimulate the production of volatile compounds as part of a metabolic response.

The calculation of rOAV > 1 enabled the identification of the key aroma contributors. Table 1 summarizes the compounds with rOAV > 1 in the *K. marxianus*/*S. cerevisiae* co-culture. Among them, 2-phenylethyl propionate was the only compound whose production decreased under co-culture conditions. This ester, formed by the reaction of 2-phenylethyl alcohol with propionic acid, contributes floral and sweet notes. Zheng et al. [19] reported that production of it by *Hanseniaspora vineae* and *Hanseniaspora osmophila* decreased in mixed fermentations with *S. cerevisiae*, consistent with the results observed in this study.

#### 3.4.1. Keys Compounds in Fermentations with *Kluyveromyces marxianus* and *Saccharomyces cerevisiae*

Among the compounds showing increased production (Table 1), several esters were notable, including benzyl acetate, ethyl octanoate, ethyl hexanoate, and ethyl decanoate. Benzyl acetate was not detected in the *K. marxianus* monoculture but was detected exclusively in co-cultures (Table S1). Although its benzyl alcohol, its direct precursor [61], was not detected, benzaldehyde was identified in *K. marxianus* monocultures (Table S1). Benzaldehyde has been proposed as a precursor to benzyl alcohol and benzyl acetate in *Cyberlindnera rhodanensis* [62], suggesting a similar biosynthetic route in the present system. Benzyl acetate has previously been reported in *S. cerevisiae* monocultures and in co-culture with *T. delbrueckii* [22], as well as in fermentations involving *Hanseniaspora vineae* and *S. cerevisiae* [63].

Ethyl esters are primarily synthesized by alcohol acetyltransferases (AATs), which catalyze the addition of an acyl group from acyl-CoA to an alcohol [49]. Ethyl octanoate, characterized by fruity notes, has been described as a key compound in various fermented beverages, including light-flavored Baijiu [64], Zhuyeqing [65], wines [66], beers, and derived spirits [67]. Higher concentrations of ethyl decanoate were in fermentations involving *S. cerevisiae* (Table S1). Zhang et al. [68] reported that ciders fermented with *S. cerevisiae* contained ethyl decanoate, whereas those fermented with *K. marxianus* did not. In contrast, the present study demonstrated that *K. marxianus* can produce this compound. Similarly, Sun et al. [69] observed that mixed fermentations of lactic acid bacteria and *K. marxianus* enhanced the production of ethyl esters, including ethyl octanoate and ethyl decanoate.

In fermentations involving *S. cerevisiae*, another group of upregulated compounds was free fatty acids, such as hexanoic, octanoic, and n-decanoic acids. These compounds have been associated with undesirable sensory descriptors, including rancid, cheesy, sweaty, and sour notes [70]. Nevertheless, they have also been reported to contribute to the body of fermented beverages and act as precursors for ester formation [71,72]. The extent to which these functions influenced the fermentations analyzed in this study remains to be determined.

Finally, terpenes contribute to desirable aroma characteristics and are often associated with β-glucosidase activity. Mixed fermentations have been reported to enhance terpene concentrations, such as nerolidol [72], which is consistent with the upregulation of nerolidol production observed in co-cultures in this study (Table 1).

The results presented here showed that the interaction between *K. marxianus* and *S. cerevisiae* was associated with higher relative abundances of nerolidol and several esters and fatty acids. These changes may enhance both the body and aromatic profile of fermented beverages. However, their actual sensory impact was not evaluated in the present study. The simultaneous increase in organic acids and esters observed in the co-cultures may reflect physiological responses to interspecific interactions. Likewise, the elevated ester production by *K. marxianus* could be consistent with stress-associated metabolic responses previously reported in yeast [73].

#### 3.4.2. Keys Compounds in Fermentations with *Kluyveromyces marxianus* and *Torulaspora delbrueckii*

The interaction between *K. marxianus* and *T. delbrueckii* resulted in decreased production of 2,4-di-tert-butylphenol, 2-furanmethyl acetate, and n-decanoic acid (Table 2), compounds commonly associated with undesirable aromas. 2,4-Di-tert-butylphenol, produced by various microorganisms, exhibits high toxicity even toward producing cells [74] and is characterized by a leather-like flavor, particularly noted in beverages such as green tea when packaged in polypropylene [75]. The reduced production of n-decanoic acid and 2,4-di-tert-butylphenol in these fermentations may be associated with improved survival of *T. delbrueckii*.

Among the upregulated compounds, nonanal (Table 2), which contributes citrus- like notes, has been previously reported in fermentations involving *T. delbrueckii* [22,61]. Nerolidol was also upregulated, contributing positively to aroma quality. In addition, geranyl acetate, a compound with a floral lavender-like scent, was detected [76]. *Torulaspora delbrueckii* is known for its ability to promote interconversion reactions among monoterpenes [18,77], which may explain the presence of this compound. These findings suggest that the interaction between *K. marxianus* and *T. delbrueckii* reduces the concentration of antimicrobial compounds, such as geraniol [78], thereby supporting coexistence between the two yeasts.

#### 3.4.3. Keys Compounds in Fermentations with *Kluyveromyces marxianus* Versus Ternary Coculture

In the ternary co-culture, several upregulated compounds, including ethyl decanoate, hexanoic acid, n-decanoic acid, octanoic acid, and nerolidol, were consistent with those observed in *K. marxianus/S. cerevisiae* fermentations (Table 3). Additionally, methionol was identified as an upregulated compound in the ternary system. Methionol contributes cooked potato-like aromas [32] and has previously been reported to be produced by *K. marxianus* under aerated conditions in whey-based media supplemented with yeast extract and peptone [79]. Yeast extract may serve as a source of methionine [53], which *S. cerevisiae* can convert into methionol via the Ehrlich pathway [80].

Future research should focus on elucidating the molecular mechanisms underlying stress-induced metabolic reprogramming in yeasts. Expanding the range of substrates and fermentation conditions evaluated would also be valuable. Such efforts could contribute to the development of predictive models for rational fermentation design, enabling the tailoring of volatile compound profiles to specific market demands, improving product consistency, and reducing off-flavors associated with stress-induced metabolic imbalance.

## 4. Conclusions

The impact of yeast competition on *K. marxianus* is strongly dependent on the nutritional composition of the culture medium used. Nutrient-rich media reduce antagonistic interactions between *S. cerevisiae* and *K. marxianus*. At the same time, nutrient-limited conditions intensify competition for scarce resources. It leads to reduced viability of *K. marxianus* and metabolic shifts that alter the fermentation volatilome. The presence of *S. cerevisiae* reduced the concentration of 2-phenylethyl propionate, while increasing the content of esters such as ethyl octanoate, ethyl hexanoate, benzyl acetate, and ethyl decanoate, as well as volatile fatty acids, including hexanoic, octanoic, and n-decanoic acids. The accumulation of these fatty acids is associated with decreased viability in *K. marxianus* and, more markedly, in *T. delbrueckii*. It should be noted that, while these compounds may influence aroma perception, sensory evaluation studies are needed to validate their effect on fermentation. Finally, future studies should include specific measurements of physiological or molecular stress indicators, such as cell membrane integrity, intracellular ROS levels, and other stress indicators. The results indicate that the volatilome of *Kluyveromyces marxianus* is not an isolated metabolic event but rather a dynamic phenomenon strongly influenced by microbial ecology and environmental conditions. Coexistence and competition with other yeasts can serve as biological triggers for the activation of secondary metabolic pathways. This physiological response redirects metabolism toward the production of key volatile compounds associated with complex aromatic profiles and high commercial value. The production of spirits such as mezcal can benefit from the design of mixed inoculants and the implementation of nutritional supplementation strategies that allow for better quality control and more accurate prediction of the sensory profile.

## Figures and Tables

**Table 1:** Relative olfactory activity (rOAV) for key compounds in samples of Km vs. Km/Sc (VIP ≥ 1, *p* < 0.05, |Log_2_(FC)| ≥ 1.0 y rOAV ≥ 1).

Compounds	Odor Descriptor	Threshold (mg/L)	M2		YPD	
			Km	Km/Sc	Km	Km/Sc
Ethyl octanoate	Sweet, floral, fruity, banana, pear, brandy ^a^	0.002 ^a^	267.182	2251.416	3616.679	10,923.973
Benzyl acetate	Sweet, floral, fruity, jasmine, fresh ^c^	0.364 ^b^	ND	0.061	ND	1.040
Ethyl Decanoate	Brandy, fruity, grape ^a^	0.5 ^a^	0.169	3.064	3.203	18.831
Ethyl hexanoate	Fruity, green apple, banana, brandy, wine-like ^a^	0.005 ^a^	101.028	231.741	998.155	2215.652
2-Phenylethyl propionate	Floral, rose red rose, fruity, honey, balsamic, storax ^c^	18 ^b^	0.333	0.092	5.020	0.606
Nerolidol	Floral, green, citrus, woody, waxy ^c^	0.25 ^b^	0.565	2.697	1.520	2.237
Hexanoic acid	Cheese, fatty ^a^	3 ^a^	0.122	0.534	0.947	1.072
Octanoic acid	Fatty, rancid ^a^	10 ^a^	0.780	2.386	2.070	4.733
n-Decanoic acid	Fatty, rancid ^a^	1 ^a^	1.452	5.855	7.359	33.981

Values are means of triplicate replicates. Km = *K. marxianus* monoculture, Km/Sc = *K. marxianus* and *S. cerevisiae* co-inoculation. ^a^ [33]. ^b^ [31]. ^c^ https://thegoodscentscompany.com.

**Table 2:** Relative olfactory activity (rOAV) for key compounds in samples of Km vs. Km/Td (VIP ≥ 1, *p* < 0.05, |Log_2_(FC)| ≥ 1.0 y rOAV ≥ 1).

Compounds	Odor Descriptor	Threshold (mg/L)	M2		YPD	
			Km	Km/Td	Km	Km/Td
2,4-Di-tert-butylphenol	Phenol ^c^	0.5 ^c^	7.010	2.662	14.536	2.465
2-Furanmethyl acetate	Toasted ^a^	0.54 ^a^	0.115	0.109	1.582	0
n-Decanoic acid	Fatty, rancid ^a^	1 ^a^	1.452	0	7.359	0
Nonanal	Citrusy, floral ^a^	0.015 ^a^	0	2.468	0	0
Nerolidol	floral, green, citrus, woody, waxy ^d^	0.25 ^b^	0.565	1.904	1.520	3.189
Geranyl acetate	Floral ^a^	0.06 ^a^	0	0	0	3.192

Values are means of triplicate replicates. Km = *K. marxianus* monoculture, Km/Td = *K. marxianus* and *T. delbrueckii* co-inoculation. ^a^ [33]. ^b^ [31]. ^c^ [30]. ^d^ https://thegoodscentscompany.com.

**Table 3:** Relative olfactory activity (rOAV) for key compounds in samples of Km vs. Km/Sc (VIP ≥ 1, *p* < 0.05, |Log_2_(FC)| ≥ 1.0 y rOAV ≥ 1).

Compounds	Odor Descriptor	Threshold (mg/L)	M2		YPD	
			Km	Km/Td	Km	Km/Td
Ethyl Decanoate	Brandy, fruity, grape ^a^	0.5 ^a^	0.169	0.984	3.203	11.086
Hexanoic acid	Cheese, fatty ^a^	3 ^a^	0.122	0.329	0.947	1.242
n-Decanoic acid	Fatty, rancid ^a^	1 ^a^	1.452	1.797	7.359	23.275
Octanoic acid	Fatty, rancid ^a^	10 ^a^	0.780	1.585	2.070	4.409
Nerolidol	floral, green, citrus, woody, waxy ^c^	0.25 ^b^	0.565	1.328	1.520	2.443
Methionol	Cooked potato-like ^d^	0.069 ^b^	1.005	3.27	17.609	25.363

Values are means of triplicate replicates. Km = *K. marxianus* monoculture, Km/Sc/Td = *K. marxianus*, *S. cerevisiae*, and *T delbrueckii* co-inoculation. ^a^ [33]. ^b^ [31]. ^c^ https://thegoodscentscompany.com. ^d^ [81].

## Data Availability

The original contributions presented in the study are included in the article/Supplementary Material, further inquiries can be directed to the corresponding author.

## Supplementary Materials

The following supporting information can be downloaded at: https://www.mdpi.com/article/10.3390/jof12070470/s1, Figure S1: Model overview. (A: Km vs. Km/Sc OPLS-DA model; B: Km vs. Km/Td OPLS-DA model; C: Km vs. Km/Sc/Td OPLS-DA model); Figure S2: Results of permutation test. (A: Km vs. Km/Sc OPLS-DA model; B: Km vs. Km/Td OPLS-DA model; C: Km vs. Km/Sc/Td OPLS-DA model); Table S1: Concentrations of volatile compounds produced by *K. marxianus* in monoculture and coculture with *S. cerevisiae*, and *T. delbrueckii*; Table S2: Overlapping compounds extracted form samples; Table S3: The relative odor activity values (rOAVs and ROAVs) for different compounds; Table S4: Statistical parameters of differential metabolites in the Km vs. Km/Sc Table S5: Statistical parameters of differential metabolites in the Km vs. Km/Td Table S6: Statistical parameters of differential metabolites in the Km vs. Km/Sc.
