# Supplementary material for: Microbial Competition and Nutrient Limitation Remodel the Volatilome of Kluyveromyces marxianus
Source: J Fungi (Basel). 2026 Jun 25;12(7):470. doi: 10.3390/jof12070470 (PMC13413099; doi:10.3390/jof12070470)
Supplement: Supplementary file 1 [file jof-12-00470-s001.zip › Table S1.pdf]

Biotic and nutritional stress induces alterations in the volatilome of *Kluyveromyces marxianus*

Table S1. Concentrations of volatile compounds produced by *K. marxianus* in monoculture and coculture with *S. cerevisiae* and *T. delbrueckii*

Compound	CAS	ERI	LRI	M2				YPD			
				Km	Km/Sc	Km/Td	Km/Sc/Td	Km	Km/Sc	Km/Td	Sc/Km/Td
**Acetals**											
1-(1-Ethoxyethoxy)pentane	13442-89-2	1108.2	1104	ND	0.06±0.009c	ND	0.026±0.003cd	0.517±0.032a	ND	ND	0.27±0.011b
Total				ND	0.06±0.009c	ND	0.026±0.003cd	0.517±0.032a	ND	ND	0.27±0.011b
**Acids**											
9-Decenoic acid	14436-32-9	2366.7	2369	ND	ND	ND	ND	ND	6.193±1.308a	ND	4.886±0.661a
Acetic acid	64-19-7	1478.9	1498	ND	0.207±0.019b	ND	0.031±0.007c	0.339±0.011a	ND	ND	0.22±0.025b
n-Decanoic acid	334-48-5	2305.9	2281	1.452±0.249d	5.855±0.394c	ND	1.797±0.08d	7.359±0.468c	33.981±0.321a	ND	23.275±2.839b
Octanoic acid	124-07-02	2077.7	2086	7.803±0.737de	23.856±2.386b	3.332±0.441e	15.854±1.265bcd	20.696±1.462bc	47.326±5.466a	15.619±1.221cd	44.091±4.847a
Isobutyric acid	79-31-2	1590.2	1595	ND	ND	ND	ND	0.334±0.055a	ND	ND	ND
Hexanoic acid	142-62-1	1885.7	1850	0.367±0.05e	1.601±0.227c	0.588±0.027de	0.988±0.033cde	2.842±0.456b	3.217±0.288ab	1.19±0.097cd	3.726±0.261a
Total				9.622±0.655ef	31.518±2.593c	3.919±0.463f	18.669±1.351d	31.569±1.794c	90.717±7.236a	16.81±1.24de	76.199±3.815b
**Alcohols**											
3-methyl-1-butanol	123-51-3	1206.7	1220	20.953±1.603c	18.919±0.572c	22.153±0.503c	13.399±0.107c	149.429±2.062a	115.466±3.085b	145.399±2.071a	124.478±9.594b
1-Decanol	112-30-1	1763.7	1752	ND	0.141±0.026a	0.122±0.011a	0.073±0.006b	ND	ND	ND	ND
1-Dodecanol	112-53-8	1968.9	1981	0.068±0.002c	0.126±0.008c	0.081±0.011c	0.066±0.006c	0.449±0.069b	0.417±0.02b	0.616±0.072a	0.384±0.055b
1-Heptanol	111-70-6	1456.5	1465	0.022±0.003e	0.326±0.023c	0.016±0.002e	0.17±0.005d	0.094±0.007de	0.899±0.09a	0.058±0.005e	0.695±0.031b
2-Ethylhexan-1-ol	104-76-7	1490.2	1504	0.042±0.004d	0.235±0.037b	0.071±0.002cd	0.143±0.022c	0.414±0.062a	ND	ND	ND
3-Methylhexan-1-ol	13231-81-7	1412.2	1413	ND	ND	ND	ND	ND	0.091±0.004c	0.126±0.009a	0.11±0.01b
1-Octanol	111-87-5	1559.2	1560	0.017±0.001f	0.111±0.004c	0.047±0.005e	0.082±0.001d	0.117±0.008c	0.222±0.006a	0.038±0.003e	0.142±0.013b
4-Methyl-1-pentanol	626-89-1	1317.7	1310	ND	ND	ND	ND	ND	0.033±0.005b	0.037±0.002ab	0.042±0.004a
2-methyl-1-Propanol	78-83-1	1087.8	1073	3.215±0.23d	1.257±0.066e	2.686±0.055d	1.064±0.031e	8.913±0.148b	5.085±0.112c	10.282±0.219a	5.411±0.533c
Farnesol	4602-84-0	2356.4	2351	ND	ND	ND	ND	1.69±0.27b	1.493±0.114b	3.23±0.243a	1.063±0.095c
5-Methylfurfuryl alcohol	3857-25-8	1730.9	1723	ND	ND	ND	ND	0.364±0.018c	0.411±0.008b	0.598±0.037a	0.359±0.021c
2-Nonanol	628-99-9	1519.4	1525	ND	ND	0.081±0.015c	ND	0.355±0.022b	0.452±0.049a	0.458±0.016a	0.405±0.034ab
2-Undecanol	1653-30-1	1720	1706	ND	ND	ND	ND	ND	ND	0.169±0.021a	ND
2-Phenylethanol	60-12-8	1924.2	1908	13.358±0.364e	46.534±1.611d	25.411±0.164e	27.496±1.383e	135.814±3.296ab	102.9±9.275c	143.567±8.589a	125.055±10.834b
Total				37.675±1.47e	67.65±1.995d	50.668±0.463de	42.493±1.328e	297.64±5.316a	227.469±10.287c	304.578±8.24a	258.144±15.144b
**Aldehydes and ketones**											
Dodecanal	112-54-9	1716.1	1716	ND	ND	0.063±0.003c	ND	0.284±0.051b	0.556±0.039a	0.263±0.038b	0.255±0.026b
Benzaldehyde	100-52-7	1535	1525	0.205±0.015c	0.501±0.042b	0.293±0.03c	0.266±0.021c	0.673±0.084a	0.625±0.071ab	0.528±0.063ab	0.614±0.046ab
p-Tolualdehyde	104-87-0	1662.8	1656	2.565±0.184a	3.059±0.269a	3.105±0.456a	3.195±0.353a	ND	ND	ND	ND
Phenylacetaldehyde	122-78-1	1657.1	1652	0.04±0.008c	0.12±0.015c	0.071±0.003c	0.082±0.002c	0.648±0.081a	0.572±0.085ab	0.484±0.067b	0.576±0.029ab
Decanal	112-31-2	1501.9	1495	0.095±0.004c	0.239±0.039a	0.214±0.012a	0.14±0.018b	ND	ND	ND	ND

Nonanal	124-19-6	1395.5	1406	ND	ND	0.037±0.003b	ND	ND	0.148±0.007a	ND	ND
3-hydroxy-2-butanone	513-86-0	1287.8	1283	ND	0.193±0.022b	ND	0.239±0.006a	0.119±0.014c	0.145±0.009c	0.133±0.008c	0.143±0.008c
Total				2.906±0.193b	4.112±0.249a	3.783±0.447a	3.923±0.316a	1.725±0.159c	2.047±0.113c	1.408±0.132c	1.589±0.043c
**Esters**											
Isoamyl propionate	105-68-0	1192.1	1188	ND	ND	0.043±0.003e	ND	0.521±0.016a	0.163±0.013d	0.468±0.015b	0.219±0.008c
Geranyl acetate	105-87-3	1762.8	1769	ND	ND	ND	ND	ND	0.588±0.076a	0.191±0.001c	0.388±0.047b
2-Furanmethyl acetate	623-17-6	1546.9	1557	0.062±0.012bc	0.151±0.031b	0.059±0.008bc	0.093±0.023bc	0.854±0.12a	ND	ND	ND
Ethyl 7-octenoate	35194-38-8	1489.5	1486	ND	ND	ND	ND	ND	ND	0.357±0.04a	0.36±0.032a
Isobutyl acetate	110-19-0	1010.6	1014	0.262±0.033a	0.051±0.008e	0.138±0.013bcd	0.059±0.006e	0.106±0.004d	0.161±0.008bc	0.121±0.004cd	0.177±0.021b
Benzyl acetate	140-11-4	1741.6	1748	ND	0.022±0.003c	0.018±0.001c	0.023±0.002c	ND	0.379±0.034a	0.214±0.026b	0.335±0.014a
Ethyl phenylacetate	101-97-3	1797.4	1776	0.085±0.016d	0.106±0.039cd	0.092±0.004d	0.063±0.009d	0.167±0.02ab	0.209±0.009a	0.156±0.013bc	0.209±0.008a
1-Phenylethyl Acetate	93-92-5	1710	1696	0.029±0.002a	ND	ND	ND	ND	ND	ND	ND
Ethyl benzoate	93-89-0	1682.3	1670	ND	ND	ND	0.031±0.003d	0.219±0.001bc	0.269±0.041ab	0.172±0.024c	0.292±0.035a
2-Phenylethyl hexanoate	6290-37-5		2165	ND	ND	ND	ND	0.211±0.025c	0.542±0.032a	0.324±0.046b	0.532±0.073a
Ethyl pyruvate	617-35-6	1280.8	1276	0.226±0.01a	0.223±0.023a	0.24±0.024a	ND	ND	ND	ND	ND
2-Phenylethyl propionate	122-70-3	1896.2	1884	5.999±0.541e	1.66±0.273f	12.838±2.109d	1.858±0.191f	90.36±0.65b	10.911±0.88d	97.87±1.979a	18.156±1.028c
Phenethyl butyrate	103-52-6	1976.2	1958	0.969±0.093de	0.273±0.073e	1.458±0.349d	0.272±0.021e	24.058±0.563a	3.507±0.562c	21.831±0.424b	3.132±0.306c
Isoamyl acetate	123-92-2	1120.5	1115	2.555±0.228d	1.859±0.225d	3.116±0.252d	1.54±0.075d	16.043±0.893b	19.245±1.014a	13.256±0.904c	19.196±0.845a
3-Phenylpropyl acetate	122-72-5	1956.2	1941	ND	ND	0.032±0.006d	ND	0.572±0.041a	0.183±0.006c	0.594±0.033a	0.329±0.028b
β-Phenethyl acetate	103-45-7	1832.2	1821	91.433±1.504e	84.223±5.316e	104.956±4.981d	67.73±0.977f	228.694±0.559a	131.714±5.068c	207.572±4.479b	132.298±5.304c
Methyl benzoate	93-58-3	1631.4	1631	ND	0.239±0.028a	0.28±0.064a	0.223±0.029a	ND	ND	ND	ND
Ethyl Decanoate	110-38-3	1641.6	1630	0.084±0.01d	1.532±0.328c	0.274±0.054d	0.492±0.026d	1.602±0.081c	9.416±0.603a	1.996±0.102c	5.543±0.355b
Ethyl Dodecanoate	106-33-2	1846.7	1850	ND	0.252±0.018c	0.073±0.007d	ND	ND	0.678±0.068a	ND	0.339±0.045b
Ethyl 9-decenoate	67233-91-4	1694.8	1688	ND	0.394±0.067de	ND	0.062±0.006f	1.412±0.145cd	7.716±0.981a	1.608±0.093c	4.278±0.514b
Ethyl acetate	141-78-6	883.95	889	39.466±1.363b	30.397±1.794c	52.036±2.19a	42.562±2.394b	27.141±1.729c	17.724±1.245d	21.205±0.237d	17.299±1.707d
Ethyl hexanoate	123-66-0	1237.4	1235	0.505±0.029c	1.159±0.146c	0.651±0.09c	0.448±0.027c	4.991±0.355b	11.078±0.551a	5.895±0.546b	10.176±1.06a
Isopentyl hexanoate	2198-61-0	1461.3	1469	ND	ND	ND	ND	0.248±0.031c	0.586±0.057a	0.36±0.054b	0.507±0.024a
Ethyl Nonanoate	123-29-5	1539.4	1541	ND	ND	ND	ND	0.099±0.007c	0.144±0.01b	0.208±0.016a	0.055±0.007d
Ethyl octanoate	106-32-1	1438.1	1431	0.534±0.039d	4.503±0.429c	0.883±0.176d	1.187±0.072d	7.233±0.7c	21.848±2.25a	13.401±1.729b	15.554±1.428b
Isoamyl decanoate	2306-91-4	1865.3	1863	ND	ND	ND	ND	ND	0.499±0.038a	0.297±0.038c	0.391±0.019b
Phenethyl isobutyrate	103-48-0	1891.5	1896	3.478±0.392b	0.699±0.057b	2.818±0.322b	0.655±0.108b	18.647±2.729a	3.715±0.439b	15.833±4.336a	2.867±0.237b
Total				145.687±0.322d	127.743±8.201de	180.006±9.289c	117.297±3.017e	423.179±2.838a	241.273±11.238b	403.93±4.911a	232.631±9.046b
**Pyrazines**											
2,5-Dimethylpyrazine	123-32-0	1324.8	1330	ND	ND	ND	ND	34.17±0.501a	35.397±2.757a	34.698±2.302a	36.155±2.737a
2,5-Dimethyl-3-(3-methylbutyl)pyrazine	18433-98-2	1661.2	1666	ND	ND	ND	ND	4.887±0.552bc	5.694±0.427ab	4.498±0.438c	5.928±0.582a

2-Ethenyl-5-methylpyrazine	13925-08-1	1501.1	1516	ND	ND	ND	ND	0.993±0.053b	1.273±0.052a	1.157±0.112a	1.246±0.038a
2-Ethenyl-6-methylpyrazine	13925-09-2	1494.7	1490	ND	ND	ND	ND	1.18±0.047a	1.255±0.071a	1.204±0.049a	1.294±0.11a
2-Ethyl-6-methylpyrazine	13925-03-6	1391.5	1395	ND	ND	ND	ND	0.393±0.048bc	0.352±0.031c	0.44±0.024ab	0.494±0.033a
2-Methyl-5-[(1Z)-1-propenyl]pyrazine	55138-66-4	1545	1535	ND	ND	ND	ND	ND	1.191±0.128a	0.925±0.083b	1.123±0.088a
3-Ethyl-2,5-dimethylpyrazine	13360-65-1	1447.3	1449	ND	ND	ND	ND	2.643±0.209c	6.994±0.414b	2.229±0.336c	8.657±0.936a
2-Methylpyrazine	109-08-0	1272.3	1257	ND	ND	ND	ND	2.548±0.019a	2.64±0.155a	2.759±0.139a	2.614±0.143a
2,3,5-Trimethylpyrazine	14667-55-1	1404	1406	ND	ND	ND	ND	0.893±0.064b	1.121±0.077a	0.907±0.012b	1.202±0.113a
Total				ND	ND	ND	ND	47.706±0.896b	55.918±3.85a	48.816±2.645b	58.713±4.103a
**Terpenes**											
Nerolidol	40716-66-3	2042.4	2055	0.141±0.027cd	0.674±0.087ab	0.476±0.123bcd	0.332±0.01de	0.38±0.076e	0.559±0.093bc	0.797±0.068a	0.611±0.082ab
Linalool	78-70-6	1549.4	1543	0.089±0.006c	0.299±0.03a	0.262±0.024ab	0.238±0.026b	0.117±0.013c	0.074±0.008c	0.089±0.007c	0.113±0.002c
Geraniol	106-24-1	1851.4	1862	0.17±0.015c	0.18±0.016c	0.294±0.032c	0.17±0.011c	2.123±0.086a	1.631±0.091b	2.382±0.232a	1.685±0.16b
Nerol	106-25-2	1805.5	1807	0.058±0.006d	0.084±0.015d	0.093±0.021d	0.062±0.001d	0.329±0.011c	0.511±0.074b	0.458±0.043	0.653±0.012a
Isogeraniol	5944-20-7	1817.2	1849	0.04±0.006d	0.062±0.002d	0.094±0.004d	0.05±0.008d	0.327±0.053c	0.492±0.04b	0.428±0.055b	0.6±0.005a
α-Terpineol	98-55-5	1701.3	1705	0.095±0.013d	0.281±0.029c	0.234±0.018c	0.233±0.013c	0.664±0.068a	0.301±0.047c	0.47±0.026b	0.435±0.008b
Geranylacetone	689-67-8	1866.8	1864	ND	0.111±0.01a	0.1±0.007a	0.098±0.005a	ND	ND	ND	ND
Citronellol	106-22-9	1768.7	1781	0.49±0.038c	0.561±0.024c	0.522±0.045c	0.397±0.034c	2.887±0.265bc	3.35±0.357ab	2.734±0.285c	3.819±0.241a
γ-geraniol	13066-51-8	1790.8	1800	0.046±0.01c	0.062±0.01c	0.087±0.006c	0.064±0.004c	0.251±0.032b	0.303±0.035b	0.288±0.022b	0.399±0.025a
Dihydromyrcenol	18479-58-8	1469.8	1470.6	0.03±0.006cd	0.055±0.009ab	0.032±0.001cd	0.06±0.006a	ND	0.027±0.003d	0.043±0.005bc	0.063±0.005a
α-Farnesene	502-61-4	1753.7	1768	ND	ND	ND	ND	0.154±0.023b	0.096±0.007c	0.213±0.015a	0.077±0.008c
Total				1.158±0.074d	2.37±0.217c	2.193±0.245c	1.705±0.085cd	7.233±0.33b	7.344±0.595b	7.901±0.033ab	8.455±0.134a
**Others**											
Phenol	108-95-2	2020.6	2008	ND	0.017±0.003c	ND	0.024±0.002c	0.051±0.002ab	0.041±0.007b	0.052±0.006ab	0.053±0.007a
5-Methylfurfural	620-02-0	1587.9	1582	ND	ND	ND	0.04±0.004a	ND	ND	ND	ND
2-Furanmethanol	98-00-0	1670.2	1685	ND	ND	ND	0.268±0.026c	1.339±0.137b	2.055±0.244a	2.084±0.157a	1.681±0.148b
2-Acetylfuran	1192-62-7	1516.6	1534	0.217±0.005d	1.149±0.217ab	0.195±0.036d	1.429±0.099a	1.031±0.047b	0.897±0.112bc	0.688±0.087c	0.902±0.082bc
Furfural	98-01-1	1477.3	1495	0.06±0.005d	0.361±0.048b	0.066±0.002d	0.09±0.004d	0.925±0.053a	0.194±0.011c	0.171±0.006c	0.187±0.028c
3-Ethoxy-1-propanol	111-35-3	1377.1	1376	ND	ND	0.021±0.003b	ND	ND	ND	0.111±0.011a	ND
δ-Dodecalactone	713-95-1	2441.1	2426	0.051±0.006c	0.056±0.006c	0.078±0.007c	0.034±0.002c	0.305±0.027a	0.211±0.036b	0.257±0.027ab	0.205±0.005b
2,4-Di-tert-butylphenol	96-76-4	2315	2312	3.505±0.136d	2.266±0.119ef	1.331±0.067f	2.54±0.271de	7.268±0.649b	5.353±0.679c	1.232±0.155f	8.676±0.618a
2-Acetylpyrrole	1072-83-9	1988.7	2003	ND	ND	ND	ND	0.338±0.042ab	0.101±0.008c	0.35±0.046a	0.271±0.025b
Methionol	505-10-2	1724.9	1723	0.069±0.012c	0.306±0.007c	0.245±0.009c	0.226±0.004c	1.215±0.062b	1.25±0.112b	2.027±0.248a	1.75±0.118a
Total				3.902±0.134d	4.155±0.38d	1.936±0.078e	4.65±0.276d	12.472±0.719a	10.1±1.027b	6.973±0.168c	13.726±0.407a

Results represent the mean ± estándar deviation in three replicates. ND: Not detected. ERI: Experimental retention index. LRI: Literature retention index. Km: *K. marxianus* , Sc: *S. cerevisiae* , Td: *T. delbrueckii* .
