# Supplementary material for: Microbial Competition and Nutrient Limitation Remodel the Volatilome of Kluyveromyces marxianus
Source: J Fungi (Basel). 2026 Jun 25;12(7):470. doi: 10.3390/jof12070470 (PMC13413099; doi:10.3390/jof12070470)
Supplement: Supplementary file 1 [file jof-12-00470-s001.zip › Table S2.pdf]

Biotic and nutritional stress induces alterations in the volatilome of Kluyveromyces marxianus

Table S2. Overlapping compounds extracted form samples

Compound	RT	RI
Ethyl butyrate	4.461	1031.2
1-Propanol	4.461	1032.4
Thymol	22.81	2186.8
Nonanoic acid	22.777	2189.6
Carvacrol	23.095	2222.6

RT: Retention time, RI: Retention index.
