# Supplementary material for: Microbial Competition and Nutrient Limitation Remodel the Volatilome of Kluyveromyces marxianus
Source: J Fungi (Basel). 2026 Jun 25;12(7):470. doi: 10.3390/jof12070470 (PMC13413099; doi:10.3390/jof12070470)
Supplement: Supplementary file 1 [file jof-12-00470-s001.zip › Table S3.pdf]

Biotic and nutritional stress induces alterations in the volatilome of Kluyveromyces marxianus

Table S3. The relative odor activity values (rOAVs and ROAVs) for different compounds.

Compound	Odor threshold (mg/L)	Odor	M2								YPD							
			Km		Km/Sc		Km/Td		Km/Sc/Td		Km		Km/Sc		Km/Td		Sc/Km/Td	
			rOAV	ROAV	rOAV	ROAV	rOAV	ROAV	rOAV	ROAV	rOAV	ROAV	rOAV	ROAV	rOAV	ROAV	rOAV	ROAV
**Acids**																		
9-Decenoic acid	4.3[c]	Waxy, green, fruity, fatty, soapy[e]	ND	ND	ND	ND	ND	ND	ND	ND	ND	ND	1.440	0.013	ND	ND	1.136	0.015
n-Decanoic acid	1[a]	Fatty, rancid[a]	1.452	0.397	5.855	0.263	ND	ND	1.797	0.304	7.359	0.206	33.981	0.314	ND	ND	23.275	0.302
Octanoic acid	10[a]	Fatty, rancid[a]	0.780	0.214	2.386	0.106	0.333	0.060	1.585	0.269	2.070	0.058	4.733	0.044	1.562	0.023	4.409	0.057
Hexanoic acid	3[a]	Cheese, fatty[a]	0.122	0.033	0.534	0.024	0.196	0.035	0.329	0.056	0.947	0.026	1.072	0.010	0.397	0.006	1.242	0.016
**Alcohols**																		
3-methyl-1-butanol	30[a]	Burnt, alcohol, nail polish, whiskey[a]	0.698	0.191	0.631	0.028	0.738	0.132	0.447	0.075	4.981	0.139	3.849	0.035	4.847	0.073	4.149	0.054
1-Dodecanol	2.47[c]	Earthy, soapy, waxy, fatty, honey, coconut[f]	0.027	0.007	0.051	0.002	0.033	0.006	0.027	0.004	0.182	0.005	0.169	0.002	0.249	0.004	0.155	0.002
1-Heptanol	2.5[b]	Green[b]	0.009	0.002	0.130	0.006	0.006	0.001	0.068	0.011	0.038	0.001	0.360	0.003	0.023	<0.001	0.278	0.004
2-Ethylhexan-1-ol	0.3[c]	Citrus, fresh, floral, oily, sweet[f]	0.139	0.038	0.784	0.035	0.236	0.042	0.476	0.080	1.381	0.038	ND	ND	ND	ND	ND	ND
1-Octanol	0.9[a]	Rose, honey[a]	0.019	0.005	0.123	0.005	0.052	0.009	0.091	0.015	0.130	0.004	0.247	0.002	0.043	0.001	0.158	0.002
2-methyl-1-Propanol	40[a]	Alcohol, nail polish[a]	0.080	0.022	0.031	0.001	0.067	0.012	0.027	0.004	0.223	0.006	0.127	0.001	0.257	0.004	0.135	0.002
Farnesol	1[a]	Floral, oily[a]	ND	ND	ND	ND	ND	ND	ND	ND	1.690	0.047	1.493	0.014	3.230	0.049	1.063	0.014
2-Nonanol	0.05[b]	Cucumber[b]	ND	ND	ND	ND	1.615	0.291	ND	ND	7.102	0.198	9.041	0.084	9.150	0.138	8.104	0.105
2-Undecanol	0.041[c]	Waxy, fatty, clean, oily, fresh, fishy, nut flesh, tallow[f]	ND	ND	ND	ND	ND	ND	ND	ND	ND	ND	ND	ND	4.114	0.062	ND	ND
2-Phenylethanol	10[a]	Rose, honey[a]	1.336	0.365	4.653	0.208	2.541	0.454	2.750	0.464	13.581	0.378	10.290	0.095	14.357	0.215	12.505	0.161
**Aldehydes and ketones**																		
Dodecanal	0.033[c]	Soapy, waxy, aldehydic, citrus, green, floral[f]	ND	ND	ND	ND	1.899	0.340	ND	ND	8.595	0.238	16.849	0.155	7.978	0.120	7.725	0.099
Benzaldehyde	5[b]	Almond, burnt sugar[b]	0.041	0.011	0.100	0.005	0.059	0.011	0.053	0.009	0.135	0.004	0.125	0.001	0.106	0.002	0.123	0.002
Phenylacetaldehyde	0.001[b]	Floral[b]	40.413	11.038	120.039	5.327	71.289	12.680	82.100	13.863	648.318	17.983	571.600	5.288	483.518	7.366	576.213	7.473
Decanal	0.001[a]	Grassy, orange skin-like[a]	95.470	26.122	239.056	10.586	214.392	38.325	140.292	23.770	ND	ND	ND	ND	ND	ND	ND	ND
Nonanal	0.015[a]	Citrusy, floral[a]	ND	ND	ND	ND	2.468	0.442	ND	ND	ND	ND	9.886	0.091	ND	ND	ND	ND
**Esters**																		
Isoamyl propionate	0.043[c]	Sweet, fruity, banana, pineapple[f]	ND	ND	ND	ND	0.994	0.178	ND	ND	12.123	0.337	3.786	0.035	10.893	0.164	5.090	0.066

Geranyl acetate	0.06[a]	Floral[a]	ND	ND	ND	ND	ND	ND	ND	ND	ND	ND	9.797	0.090	3.192	0.048	6.462	0.084
2-Furanmethyl acetate	0.54[a]	Toasted[a]	0.115	0.031	0.280	0.013	0.109	0.020	0.172	0.029	1.582	0.044	ND	ND	ND	ND	ND	ND
Isobutyl acetate	1.6[a]	Banana, fruity, sweet[a]	0.163	0.045	0.032	0.001	0.086	0.016	0.037	0.006	0.066	0.002	0.100	0.001	0.076	0.001	0.111	0.001
Benzyl acetate	0.364[c]	Sweet, floral, fruity, jasmi n, fresh[f]	ND	ND	0.061	0.003	0.049	0.009	0.062	0.011	ND	ND	1.040	0.010	0.587	0.009	0.919	0.012
Ethyl phenylacetate	0.04[b]	Mint[b]	2.133	0.583	2.648	0.117	2.309	0.412	1.569	0.264	4.184	0.117	5.216	0.048	3.910	0.059	5.220	0.068
1-Phenylethyl Acetate	0.16[c]	Green leafy gardenia rhub arb musty[f]	0.179	0.049	ND	ND	ND	ND	ND	ND	ND	ND	ND	ND	ND	ND	ND	ND
2-Phenylethyl propionate	18[c]	Floral, rose red rose, fruity, honey, balsa mic, storax[f]	0.333	0.091	0.092	0.004	0.713	0.128	0.103	0.017	5.020	0.140	0.606	0.006	5.437	0.082	1.009	0.013
Isoamyl acetate	0.03[a]	Sweet, fruity, apple, banana[a]	85.174	23.297	61.965	2.760	103.866	18.637	51.335	8.688	534.764	14.827	641.503	5.907	441.868	6.662	639.880	8.283
3-Phenylpropyl acetate	0.1[c]	Sweet, balsamic, storax, s picy, cinnamon[f]	ND	ND	ND	ND	0.317	0.056	ND	ND	5.719	0.160	1.832	0.017	5.943	0.089	3.288	0.042
β-Phenethyl acetate	0.25[a]	Flowery[a]	365.732	100.000	336.891	14.999	419.824	75.341	270.920	45.759	914.777	25.459	526.855	4.855	830.287	12.525	529.193	6.836
Methyl benzoate	0.00052[d]	Phenol, wintergreen, almond, floral, canga[d]	ND	ND	458.957	20.352	539.045	94.271	428.560	72.035	ND	ND	ND	ND	ND	ND	ND	ND
Ethyl Decanoate	0.5[a]	Brandy, fruity, grape[a]	0.169	0.046	3.064	0.135	0.549	0.098	0.984	0.166	3.203	0.089	18.831	0.173	3.991	0.060	11.086	0.144
Ethyl Dodecanoate	1.5[a]	Candy, floral, waxy, soap[a]	ND	ND	0.168	0.008	0.049	0.009	ND	ND	ND	ND	0.452	0.004	ND	ND	0.226	0.003
Ethyl acetate	7.5[a]	Pineapple, fruity, solvent, balsamic[a]	5.262	1.440	4.053	0.180	6.938	1.245	5.675	0.959	3.619	0.101	2.363	0.022	2.827	0.043	2.307	0.030
Ethyl hexanoate	0.005[a]	Fruity, green apple, banana, brandy, wine-like[a]	101.028	27.634	231.741	10.427	130.149	23.530	89.531	15.152	998.155	27.874	2215.652	20.394	1179.015	17.710	2035.292	26.153
Isopentyl hexanoate	0.32[d]	Fruity, banana, apple, pineapple, green[d]	ND	ND	ND	ND	ND	ND	ND	ND	0.774	0.022	1.830	0.017	1.125	0.017	1.584	0.020
Ethyl Nonanoate	1.3[a]	Fruity, floral[a]	ND	ND	ND	ND	ND	ND	ND	ND	0.076	0.002	0.110	0.001	0.160	0.002	0.042	0.001
Ethyl octanoate	0.002[a]	Sweet, floral, fruity, banana, pear, brandy[a]	267.182	73.012	2251.416	100.000	441.384	80.066	593.444	100.000	3616.679	100.000	10923	100.000	6700.739	100.000	7776.883	100.000
Isoamyl decanoate	5[c]	Waxy banana fruity sweet cognac green[f]	ND	ND	ND	ND	ND	ND	ND	ND	ND	ND	0.100	0.001	0.059	0.001	0.078	0.001
**Pyrazines**																		
2,5-Dimethylpyrazine	17[c]	cocoa, roasted, nutty, bee fy[f]	ND	ND	ND	ND	ND	ND	ND	ND	2.010	0.056	2.082	0.019	2.041	0.031	2.127	0.027

2-Ethyl-6-methylpyrazine	0.04[c]	Roasted, hazelnut, nutty[f]	ND	ND	ND	ND	ND	ND	ND	ND	9.822	0.275	8.810	0.082	10.989	0.165	12.346	0.160
3-Ethyl-2,5-dimethylpyrazine	0.0086[d]	Potato, cocoa, roasted, nutty[d]	ND	ND	ND	ND	ND	ND	ND	ND	307.338	8.579	813.278	7.527	259.169	3.955	1006.665	13.014
2,3,5-Trimethylpyrazine	0.4[c]	nutty, nut skin, earthy, cocoa, potato, peanut[f]	ND	ND	ND	ND	ND	ND	ND	ND	2.231	0.062	2.802	0.026	2.267	0.034	3.004	0.039
**Terpenes**																		
Nerolidol	0.25[c]	Floral, green, citrus, wood y, waxy[f]	0.565	0.154	2.697	0.120	1.904	0.343	1.328	0.224	1.520	0.042	2.237	0.021	3.189	0.048	2.443	0.031
Linalool	0.015[b]	Flower, lavender[b]	5.952	1.627	19.946	0.886	17.439	3.099	15.860	2.690	7.804	0.216	4.926	0.045	5.940	0.090	7.555	0.098
Geraniol	0.03[a]	Roses, geranium[a]	5.666	1.551	6.014	0.268	9.789	1.761	5.682	0.962	70.765	1.973	54.362	0.503	79.386	1.188	56.179	0.723
Nerol	0.049[d]	Lemon, fresh[d]	1.177	0.322	1.721	0.076	1.905	0.344	1.269	0.215	6.713	0.186	10.422	0.097	9.341	0.140	13.317	0.172
α-Terpineol	0.3[d]	Pine, iris, teil[d]	0.315	0.086	0.938	0.042	0.780	0.138	0.777	0.131	2.214	0.061	1.004	0.009	1.566	0.024	1.449	0.019
Citronellol	0.1[a]	Green lemon[a]	4.899	1.340	5.606	0.250	5.215	0.936	3.968	0.672	28.872	0.808	33.501	0.311	27.336	0.416	38.192	0.495
**Others**																		
Phenol	0.03[d]	phenol, medicinal[d]	ND	ND	0.563	0.025	ND	ND	0.810	0.137	1.696	0.047	1.359	0.013	1.736	0.026	1.775	0.023
2-Furanmethanol	4.5005[c]	Alcoholic, sweet, caramellic, bready , coffee[f]	ND	ND	ND	ND	ND	ND	0.060	0.010	0.298	0.008	0.457	0.004	0.463	0.007	0.373	0.005
δ-Dodecalactone	0.098[c]	Fresh sweet metallic peach oily coconut buttery[e]	0.523	0.143	0.573	0.026	0.794	0.143	0.350	0.059	3.114	0.087	2.150	0.020	2.627	0.040	2.092	0.027
2,4-Di-tert-butylphenol	0.5[d]	Phenol[d]	7.010	1.917	4.531	0.202	2.662	0.476	5.079	0.861	14.536	0.405	10.706	0.099	2.465	0.037	17.353	0.225
Methionol	0.069[c]	Cooked potato-like[h]	1.005	0.275	4.442	0.198	3.545	0.634	3.270	0.552	17.609	0.490	18.116	0.168	29.383	0.439	25.363	0.327

Results represent the mean ± estándar deviation in three replicates. ND: Not detected. Km: *K. marxianus* , Sc: *S. cerevisiae* , Td: *T. delbrueckii* .

[a] Wei et al., 2020

[b] Wang et al., 2023

[c] Van Gemert 2011

[d] Liang et al., 2024

[e] Ma et al., 2022

[f] https://thegoodscentscompany.com

[g] Chen et al., 2023

[h] Wang et al., 2023b
