# Supplementary material for: Microbial Competition and Nutrient Limitation Remodel the Volatilome of Kluyveromyces marxianus
Source: J Fungi (Basel). 2026 Jun 25;12(7):470. doi: 10.3390/jof12070470 (PMC13413099; doi:10.3390/jof12070470)
Supplement: Supplementary file 1 [file jof-12-00470-s001.zip › Table S4.pdf]

Biotic and nutritional stress induces alterations in the volatilome of Kluyveromyces marxianus

Table S4. Statistical parameters of differential metabolites in the Km vs Km/Sc

Compounds	VIP	P-value	Fold_Change	Log2FC	Type
Ethyl Dodecanoate	1.89	9.5E-12	55.157	5.7855	Up
1-Heptanol	1.88	1.3E-10	12.476	3.6411	Up
1-Octanol	1.82	1.7E-06	3.5957	1.8463	Up
Phenethyl isobutyrate	1.8	4E-06	0.21989	-2.1852	Down
Octanoic acid	1.77	1.3E-05	2.7305	1.4491	Up
Hexanoic acid	1.68	0.00023	2.2317	1.1582	Up
Ethyl octanoate	1.63	0.00031	4.6597	2.2203	Up
2-methyl-1-Propanol	1.63	0.00049	0.49589	-1.0119	Down
Ethyl Decanoate	1.59	0.00067	8.9708	3.1652	Up
n-Decanoic acid	1.57	0.00078	4.8391	2.2748	Up
3-hydroxy-2-butanone	1.39	0.00779	6.3217	2.6603	Up
2-Phenylethyl propionate	1.33	0.00947	0.17261	-2.5345	Down
Benzyl acetate	1.3	0.01282	17.906	4.1624	Up
Nerolidol	1.28	0.01771	3.1864	1.6719	Up
Ethyl 9-decenoate	1.16	0.03163	7.9537	2.9916	Up
2-Acetylfuran	1.2	0.03182	2.6702	1.4169	Up
Phenethyl butyrate	1.15	0.03357	0.19622	-2.3494	Down
Ethyl hexanoate	1.12	0.04063	2.6044	1.3809	Up
Geranylacetone	1.11	0.04948	12.551	3.6497	Up
Isoamyl decanoate	1.08	0.04948	20.81	4.3792	Up
3-Methylhexan-1-ol	1.08	0.04962	4.2053	2.0722	Up
2-Methyl-5-[(1Z)-1-propenyl]pyrazine	1.08	0.04985	48.946	5.6131	Up

Q2	0.923
R2Y	0.823
Permutacion 1000	
Q2	0.986
p	0.002
R2Y	0.988
p	0.001
