# Supplementary material for: Microbial Competition and Nutrient Limitation Remodel the Volatilome of Kluyveromyces marxianus
Source: J Fungi (Basel). 2026 Jun 25;12(7):470. doi: 10.3390/jof12070470 (PMC13413099; doi:10.3390/jof12070470)
Supplement: Supplementary file 1 [file jof-12-00470-s001.zip › Table S5.pdf]

Biotic and nutritional stress induces alterations in the volatilome of Kluyveromyces marxianus

Table S5. Statistical parameters of differential metabolites in the Km vs Km/Td

Compounds	VIP	P-value	Fold_Change	Log2FC	Type
n-Decanoic acid	2.174	2.6E-08	0.0027554	-8.5035	Down
3-Ethoxy-1-propanol	2.101	5.6E-06	5.0915	2.3481	Up
2,4-Di-tert-butylphenol	1.759	0.00115	0.26849	-1.8971	Down
Benzyl acetate	1.758	0.00321	7.7201	2.9486	Up
Nerolidol	1.626	0.00515	2.5456	1.348	Up
2-Furanmethyl acetate	1.657	0.01071	0.19673	-2.3457	Down
Furfural	1.474	0.03121	0.34408	-1.5392	Down
Geranyl acetate	1.349	0.0494	5.9355	2.5694	Up
Acetic acid	1.376	0.04947	0.10247	-3.2868	Down
Geranylacetone	1.196	0.04967	9.7512	3.2856	Up
1-(1-Ethoxyethoxy)pentane	1.373	0.0499	0.068392	-3.87	Down
Nonanal	1.196	0.04993	3.9225	1.9718	Up
Ethyl Dodecanoate	1.19	0.04996	7.2539	2.8588	Up
2-Methyl-5-[(1Z)-1-propenyl]pyrazine	1.351	0.04999	26.719	4.7398	Up

Q2	0.736
R2Y	0.896
Permutacion 1000	
Q2	0.968
p	0.003
R2Y	0.99
p	0.006
