# Supplementary material for: Microbial Competition and Nutrient Limitation Remodel the Volatilome of Kluyveromyces marxianus
Source: J Fungi (Basel). 2026 Jun 25;12(7):470. doi: 10.3390/jof12070470 (PMC13413099; doi:10.3390/jof12070470)
Supplement: Supplementary file 1 [file jof-12-00470-s001.zip › Table S6.pdf]

Biotic and nutritional stress induces alterations in the volatilome of Kluyveromyces marxianus

Table S6. Statistical parameters of differential metabolites in the Km vs Km/Sc/Td

Compounds	VIP	P-value	Fold_Change	Log2FC	Type
1-Heptanol	1.96	5.9E-10	8.8042	3.1382	Up
Phenethyl isobutyrate	1.89	1.7E-06	0.19733	-2.3413	Down
Octanoic acid	1.84	1.2E-05	2.3738	1.2472	Up
Hexanoic acid	1.77	5.2E-05	2.0766	1.0543	Up
Phenol	1.54	0.00175	2.3448	1.2294	Up
1-Octanol	1.56	0.00178	2.8849	1.5285	Up
Nerolidol	1.51	0.00319	2.3172	1.2124	Up
Benzyl acetate	1.49	0.00523	11.412	3.5125	Up
Ethyl Decanoate	1.41	0.00989	4.7063	2.2346	Up
3-hydroxy-2-butanone	1.29	0.01766	8.6104	3.1061	Up
2-Phenylethyl propionate	1.33	0.01963	0.27052	-1.8862	Down
2-Furanmethanol	1.24	0.02677	2.4234	1.2771	Up
Phenethyl butyrate	1.26	0.03046	0.18386	-2.4433	Down
Metionol	1.2	0.03467	2.1331	1.093	Up
n-Decanoic acid	1.22	0.03497	2.7998	1.4853	Up
2-Acetylfuran	1.16	0.03821	3.8385	1.9405	Up

Q2	0.811
R2Y	0.923
Permutacion 1000	
Q2	0.985
p	0.006
R2Y	0.998
p	0.006
