# Supplementary material for: Microbial Competition and Nutrient Limitation Remodel the Volatilome of Kluyveromyces marxianus
Source: J Fungi (Basel). 2026 Jun 25;12(7):470. doi: 10.3390/jof12070470 (PMC13413099; doi:10.3390/jof12070470)
Supplement: Supplementary file 1 [file jof-12-00470-s001.zip › Figure S1.pdf]

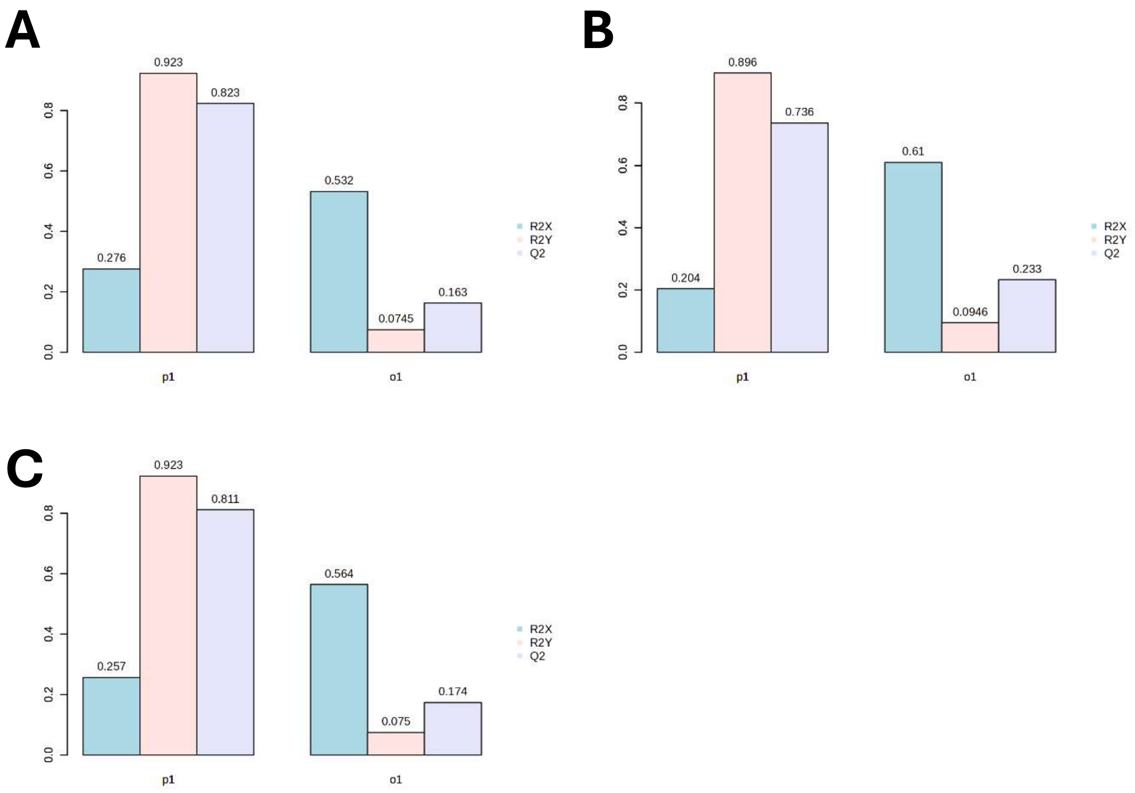

Figure S1. Model overview. (A: Km vs. Km/Sc OPLS-DA model; B: Km vs. Km/Td OPLS-DA model; C: Km vs. Km/Sc/Td OPLS-DA model).
